# The sitting position during neurosurgical procedures does not influence serum biomarkers of pulmonary parenchymal injury

**DOI:** 10.1186/1471-2482-12-24

**Published:** 2012-12-05

**Authors:** Izabela Duda, Konstancja Grzybowska, Halina Jędrzejowska-Szypułka, Joanna Lewin-Kowalik

**Affiliations:** 1 Department of Anesthesiology and Intensive Care, Medical University of Silesia, Medykow 14, Katowice, 40-75, Poland; 2 Department of Physiology, Medical University of Silesia, Medykow 18, Katowice, 40-752, Poland

**Keywords:** Sitting position, Surfactant protein D, Clara cell protein, Air embolism

## Abstract

**Background:**

The sitting position during neurosurgical operations predisposes to air penetration through veins and the movement of the air through the pulmonary circulation. Contact of an air bubble with the endothelium can lead to acute lung injury. The presence of specific pulmonary proteins in the plasma such as surfactant protein D (SP-D) and Clara cell protein (CC16) is a biomarker of damaging processes at the air-blood barrier. The aim of our study was to examine the hypothesis that the level of investigated pulmonary biomarkers in plasma is higher in patients operated on in the sitting position.

**Methods:**

The study included patients undergoing planned neurosurgical operations, who were divided into two groups: the sitting group (40 patients, operated on in the sitting position) and the supine group (24 patients, operated in the supine position). After the operation blood samples were drawn, centrifuged, frozen and stored until analyses were conducted. The determination of the SP-D and CC16 levels was performed using an ELISA test. Air embolism (VAE) was defined as a sudden drop in etCO_2_ of more than 2 mmHg and the presence of air bubbles in the aspirated blood from the central cannula. In all patients, the number of hospitalization days in the postoperative period was calculated.

**Results:**

There were no differences in the average levels of SP-D between the groups (the mean in the sitting group was 95.56 ng/mL and the mean in the supine group was 101.21 ng/mL). The average levels of CC16 were similar in both groups as well (6.56ng/mL in the sitting group and 6.79ng/mL in the supine group). There was a statistically significant positive correlation between SP-D and CC16 values in both groups. VAE was diagnosed clinically in 12.5% of cases in the sitting group without a significant increase in SP-D and CC16 levels. On average, patients in both groups were discharged from the hospital within 9 days of surgery.

**Conclusion:**

The sitting position and intraoperative VAE during neurosurgical procedures do not affect the concentration of plasma biomarkers of pulmonary parenchymal injury such as SP-D and CC16.

## Background

In 1821, Magendie published the first report of intra-operative pulmonary air embolism. The case occurred during a procedure to remove a tumor from the neck carried out in the sitting position. The patient did not survive the operation [1]. Since then the challenges and potential complications of the sitting position in neurosurgery have been widely discussed, especially with regard to intraoperative air embolism [2-5].

Placing the patient in the sitting position during neurosurgical procedures when the operative site lies above the right atrium of the heart predisposes to air entrainment through damaged vein. The incidence of air embolism (diagnosis) in these cases ranges from 5% to 83%, depending on the method of air detection [6,7].

When large volumes of air are rapidly entrained, the diagnosis of air embolism can be made easily. Obstruction of pulmonary venous blood flow by air bubbles increases right atrial and pulmonary artery pressures and the work of the right ventricle. This situation leads to a decrease in cardiac preload, a reduction in cardiac output and cardiovascular depression. However, the most common form of venous air embolism has a more insidious character. A series of air bubbles similar to a string of pearls enters the venous circulation is then carried through the right atrium, right ventricle and pulmonary artery to the lungs. In the pulmonary circulation, lipid particles, fibrin fibres and platelet aggregates are deposited on gas bubbles, and as a consequence inflammatory mediators and vasoconstricting factors are released. The mechanical contact of the gas bubble with endothelium results in increased vascular permeability. Histologically, thickening of the lung interstitium between the pulmonary alveolar basement membrane and capillary endothelium is evident. Inflammatory cells penetrate the interstitium and alveoli damaging the endothelium and provoking acute lung injury [8].

The lung epithelium produces a complex array of secretions, including surfactant and several proteins important for protective immunity and lung function. Some of these proteins are present in small quantities in the blood as well as the bronchoalveolar fluid. As these proteins are mainly, if not exclusively, secreted within the airways their appearance in the vascular compartment is assumed to result from leakage from the lungs into the blood.

Thus, they can be used as biomarkers of lung injury and the presence of these proteins in the blood reflects the extent of alveolar dysfunction at the air-blood barrier [9,10].

Specific endogenous lung proteins, such as surfactant proteins A and B (SP-A and SP-B) were first detected in the blood of children with respiratory distress syndrome (RDS) by Chida and colleagues [11] Later, Bernard and colleagues reported the presence of Clara cell protein in plasma [12], and since then interest in the field has grown considerably, reflecting the scientific and clinical importance of identifying biomarkers of respiratory pathophysiology [13-15]. The most important markers detected in plasma of patients with lung injury are those specific to the epithelium, namely SP-A and surfactant protein D (SP-D), Krebs von den Lungen mucin (KL-6), Clara cell protein (CC16 ), cytokeratin (CK19), carbohydrate antigen (CA 19–9) and carbohydrate antigen sialyl Lewis (SLX) [16].

SP-D plays a role in immune defence by acting as an opsonin to enhance phagocytosis and appears to be the protein whose concentration is best correlated with the extent of pulmonary parenchymal damage [10].

CC-16 is a low molecular weight protein secreted mainly into the airways by non-ciliated Clara cells. Although its function is not known, the concentration of CC16 in the plasma is increased in certain diseases caused by damage to the air-blood barrier [10].

We hypothesized that the air microbubbles that enter the venous system during neurosurgery in the sitting position increase the levels of SP-D and CC16 in the blood that the extend of venous air embolism correlates with the concentration of these biomarkers.

## Methods

The study was approved by the Bioethics Committee. Written informed consent was obtained from all patients. Patients scheduled for elective intracranial operations with an estimated duration of 4 h or more were consecutively enrolled in this prospective observational study. Patients were divided into two groups those who underwent craniectomy for pathology in the posterior fossa and a control group who underwent surgery in the supine position for supratentorial lesions. Patients with a history of pulmonary disease (chronic obstructive pulmonary disease, pneumonia, pulmonary embolism, pulmonary fibrosis, previous lung resection or lung tumour), immunosuppressed patients, pregnant women and patients with serious comorbidities (American Society of Anesthesiologists status IV and V) were excluded from this study.

All patients were anesthetised and ventilated according to the following protocol. Anaesthesia was induced with of propofol (6–12 mg kg^-1^) and remifentanil (0.5-0.9 mcg kg^-1^). Cisatracurium was used to facilitate intubation and to maintain neuromuscular blockade. Anaesthesia was maintained with propofol (3-5mg kg^-1^) and remifentanil (0.07-0.13 mcg kg^-1^ min^-1^) infusions. After endotracheal intubation, patients were machanically ventilated using intermittent positive pressure (IPPV) with low tidal volumes (8 ml kg^-1^ ideal body weight). The fraction of inspired oxygen (FiO_2_) was set at 40% and the inhalation/exhalation ratio at 1:2. The ventilator frequency was adjusted to maintain moderate hyperventilation (etCO_2_ 35–40 mmHg). A central cannula was placed in the subclavian vein of all patients, and position in the right atrium area was confirmed using the electrocardiogram.

In the event of a sudden drop in etCO_2_ of more than 2 mmHg, blood was aspirated from the subclavian catheter [3]. The presence of air bubbles led to the diagnosis of venous air embolism.

Information regarding the number of days of hospitalization after the surgery was collected for all of the patients.

To determine the values of SP-D and CC16 blood samples were collected immediately after surgery from the venous catheter and immediately centrifuged at 1500 rpm for 10 minutes. The supernatant was frozen and stored at −50°C until the analysis , which was performed a sandwich-type enzyme-linked immunosorbent assay (ELISA) technique using surfactant Protein D Human ELISA (RD194059101, Biovendor, Czech Republic) and Clara Cell Protein Human ELISA (RD191022200, Biovendor, Czech Republic) kits. All assays were performed in duplicate by an investigator blinded to the patients’ clinical data.

### Statistical analysis

Quantitative variables are presented as number, mean, standard deviation (SD), median, minimum and maximum values.

The normality of the distribution of CC-16 and SP-D levels was estimated using the Shapiro-Wilk test. The chi-squared and Fisher’s exact tests were used to compare quantitative variables. For normally distributed data, the Student’s t-test was used to examine differences between groups; the Mann–Whitney U test was used for data that were not normally distributed. Spearman’s correlation coefficient was employed to estimate the incidence and strength of the relationship between two quantitative variables. The research hypotheses were verified by Levene’s test. In all analyses a significance level of p < 0.05 was adopted. All analyses were performed with SPSS Statistics 17.0 software.

## Results

Between August 2010 and June 2011, 64 patients were enrolled in the study and divided into two groups. Forty patients in the study group had surgery in the sitting position (“SITTING group”) and twenty-four patients in the control group underwent surgery in the supine position (“SUPINE group”). The basic characteristics of all patients are shown in Table 1.

**Table 1:** Basic characteristic

	**SITTING**	**SUPINE**	**p-value**
	**n=40**	**n=24**	
	**(mean ± SD)**	**(mean ± SD)**	
age (years)	50.5 ± 13.8	47.0 ± 14.2	0.402
gender (M / F)	24/16	13/11	0.794
body weight (kg)	75.5 ± 13.6	77.71 ± 11.9	0.432
ASA	1.95 ± 0.69	2.25 ± 0.79	0.085
operation time (min)	279.37 ± 69.5	221.67 ± 75.5	0.003*
SBP (mmHg)	132.50 ± 19.2	124.08 ± 17.1	0.084
DBP (mmHg)	75.07 ± 13.3	77.17 ± 12.8	0.528
HR (beap / min)	80.50 ± 13.1	73.54 ± 12.8	0.036*
Saturation (%)	96.93 ± 2.3	97.42 ± 1.6	0.351
Type of operation			
cerebellopontine angle tumor	26	0	
metastatic tumor	3	0	
trigeminalgia	1	0	
meningioma	2	7	
primary tumor	3	14	
cyst	3	0	
Arnold Chiari	2	0	
angioma	0	3	

* statistically significant.

**Figure 1:**
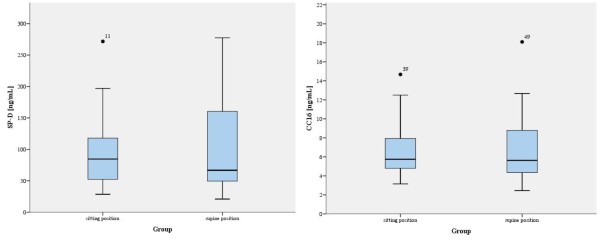
**Surfactant protein D (SP-D) and Clara cell protein (CC16) concentration in patients undergoing surgery in the sitting and the supine positions.** The horizontal line represents the median; the box encompasses 25th - 75th percentiles (the interquartile range, IQR), and the whiskers show upper and lower quartiles.

The mean serum concentration of SP-D in the SITTING group ranged from 28 to 271 ng/mL with mean of 95.56 ng/mL ±54.01 (mean ± SD) and it was similar to those observed in the SUPINE group (range 20–277 ng/mL; mean 101.21 ng/mL ± 73.16). There were also no statistically significant difference in CC16 levels between the groups (p = 0.857, median concentration in SITTING group: 5.75 ng/mL; median concentration in SUPINE group: 5.64 ng/mL). The range of values in the SITTING group was 3–14 ng/mL (mean 6.56 ng/mL ± 2.69 and 2–18 ng/mL ( mean 6.79 ng/mL ± 3.65) in the SUPINE group (Figure 1).

**Figure 2:**
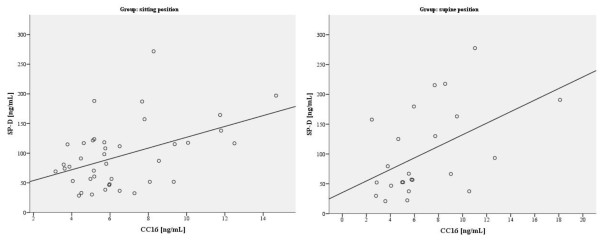
Correlation between surfactant protein D (SP-D) and Clara cell protein (CC16) concentration in the sitting and the supine position groups.

A statistically significant, positive relationship between the values of SP-D and CC16 in both the SITTING group (p = 0.029) and SUPINE group (p = 0.009) was found. If the levels of one marker increased, the levels of the other also increased. The value of the correlation coefficient for the above relationship was 0.345 in the SITTING group and 0.522 in the SUPINE group (Figure 2).

VAE was diagnosed on the basis of a decrease in etCO_2_ and confirmed by aspiration of air from the central cannula on 5 occasions in the SITTING group (12.5%) but was not diagnosed on any occasion in the SUPINE group. During VAE, etCO_2_ fell on average by 10.6 mmHg ( range 6–20 mmHg). We did not see any evidence of cardiovascular depression in the form of changes in blood pressure, cardiac arrhythmias or decreases in peripheral blood oxygen saturation in any patient diagnosed with VAE. The duration of surgery was, on average, 54 minutes longer when VAE was diagnosed (p <0.001).

**Figure 3:**
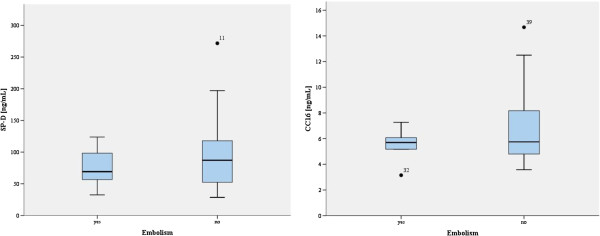
**Surfactant protein D (SP-D) and Clara cell protein (CC16) concentration in patients operated in the sitting position with vs. without intraoperative air embolism (VAE).** The horizontal line represents the median; the box encompasses the 25th - 75th percentiles (the interquartile range, IRQ), and the whiskers show upper and lower quartiles.

In the subgroup of patients with VAE, there were no statistically significant differences in the concentrations of SP-D (p = 0.513) or CC16 (p = 0.561) compared with patients without VAE. The median plasma concentrations of SP-D and CC16 were 69.19 ng/mL and 5.70 ng/mL respectively (Figure 3).

Patients from both groups were discharged from the hospital on average approximately 10 days after the surgery (p = 0.462; median 9 days for SITTING and SUPINE).

## Discussion

The main objective of this study was to determine whether there is a difference between plasma levels of SP-D and CC16 in patients undergoing neurosurgery in the sitting position compared with patients operated on in the supine position. We found that the mean concentrations of SP-D and CC16 were similar in both groups. It is well recognised that the intraoperative sitting position is a situation that can result in iatrogenic penetration of air into the venous system and pulmonary circulation. Even in situations when a large air embolism was not diagnosed intraoperatively, a gradual permeation of air microbubbles can obstruct blood flow in distal capillaries. Reduced blood flow causes tissue ischaemia, and the microbubbles initiate an immediate inflammatory response and complement activation. VAE incidents have been detected by transesophageal echocardiography (TEE) in 76% of patients undergoing surgery in the sitting position [17]. It is therefore reasonable to assume that air microbubbles are present to a certain extent in the vast majority of cases. The lung injury and endothelial dysfunction that air bubbles cause have been confirmed in animal studies [18], although in one study that used electron microscopy no physical damage to the endothelium could be visualised [19], Endothelial cells normally have tight connections to prevent leakage of fluids into the surrounding tissues. Air bubbles in the microcirculation cause pressure on endothelial cells and increase the pore radius.

The appearance of gaps between endothelial cells in leakage of fluid and consequently interstitial pulmonary oedema [20]. In our study we did not confirm potential lung injury based on the increased levels of pulmonary proteins in the systemic circulation. Furthermore, we did not observe any differences in the duration of postoperative hospitalisation or the incidence of clinical symptoms or signs between the groups.

A small amount of SP-D can be detected in the blood of healthy individuals; the exact quantity is genetically determined and varies between individuals. Plasma levels of these proteins can be elevated in patients with a wide variety of diseases and after exposure to toxins. Determann and colleagues observed SP-D levels of 140 ng/mL in patients without lung injury, which almost doubled toh acute lung injury / acute respiratory distress syndrome (ALI/ARDS) [21]. However, a different study found the mean value of SP-D to be 88 ng/mL ( range 1–1354 ng/mL) in patients with lung injury. [15] In healthy adults and children the average level of SP-D is approximately 60 ng/mL. [10] Increased levels of SP-D have been reported in idiopathic pulmonary fibrosis (IFP), tuberculosis, pulmonary alveolar proteinosis, andlues vave ranger between 100 ng/mL (sarcoidosis) to 339 ng/mL (IPF) compared to a control group of healthy patients (66ng/mL). However, SP-D levels do not appear to be elevated in asthma, bacterial pneumonia, emphysema, bronchitis or bronchiectasis [10,22]. In our study, the SP-D level in healthy, mechanically ventilated patients during craniotomy without prior lung injury was 100 ng/mL. Mechanical ventilation can lead to lung damage, in an animal model the consequences of ventilator-associated lung injury (VALI) are increased pulmonary capillary permeability and pulmonary oedema, cellular damage and necrosis and d*iffuse alveolar damage*. The factors affecting the development of VALI are positive end expiratory pressure (PEEP), the respiratory rate, pulmonary artery pressure, the arterial partial pressure of CO_2_, beta-adrenergic agonists and body position [23]. Determann and colleagues. studied the effect of ventilation on the levels ofSP-A, SP-D and CC16 and found that levels did not differ in patients mechanically ventilated with low tidal volumes and PEEP compared with a conventional ventilation technique in participants undergoing surgery in the supine position [24]. In contrast, lung damage is significantly lower in patients ventilated in the prone position compared with supine patients. [25] Similarly, the sitting position appears to improve ventilation parameters [26]. However, these observations were made in patients who were not a risk of VAE.

The plasma CC16 concentration depends on factors such as lipid levels, body mass index, gender and smoking status, and changes can be observed in various lung diseases and after exposure to toxins. A decrease in the CC16 concentration was reported, for example, in smokers, while in patients with interstitial lung diseases an increase has been observed [27,28]. The plasma concentration of CC16 in healthy subjects was determined to be between 11.8 and 27.9 ng/mL [29,30]. CC16 is a small molecule (16 kDa) and its presence in the blood is a sensitive marker of alveolar damage [22]. However, in our study CC16 concentrations were similar in both groups.

Air embolism was diagnosed in 12.5% of cases. The incidence of intraoperative diagnosis of VAE is dependent on the method of detection. The more sensitive the method is the higher the incidence of VAE [31]. We diagnosed VAE on the basis of a sudden fall of >2 mmHg in end tidal CO_2_ (etCO_2_) and confirmation of the presence of air in blood drawn from the central venous catheter. This method is capable of detecting large embolisms, which can lead to lung injury. In our study, potential lung damage was not confirmed on the basis the changes detected in our chosen biomarkers. VAE is a dynamic condition in which embolic air diffuses across the alveolar capillaries and can thus be expelled in approximately 30 minutes [32]. Short-term resistance to flow in the pulmonary capillaries does not necessarily activate the inflammatory cascades that lead to a complex mechanism that results in the contact of air bubbles with the endothelium, leading to interstitial pulmonary oedema and then ALI/ARDS.

There were also no apparent clinical consequences of lung damage in patients operated in the sitting position, as the duration of hospital stay after surgery was not prolonged.

One limitation of our study is that we only took a blood sample once after surgery. Sampling within four hours of placing the patients in the sitting position could have missed later significant lung damage and thus a later peak of blood biomarkers. More frequent sampling could have revealed the time course of changes in biomarkers rather than providing a snapshot. Another limitation may be our choice of biomarkers and the fact that differences may have been observed if other proteins or cell-specific markers of acute lung injury and acute inflammation had been measured.

## Conclusion

In conclusion, we did not find any evidence of lung damage in patients undergoing neurosurgery in the sitting position. Our choice of biomarkers (SP-D and CC16) did not allow us to definitively identify whether microbubbles of air passing into the circulatory system lead to pulmonary endothelial damage. Therefore, our understanding of the pathophysiological processes of lung injury provoked by VAE is not complete and requires further study.

## Competing interests

The authors declare no conflicts of interest.

## Authors’ contributions

ID: formulated the hypothesis, coordinated the study, evaluated data and conceived the manuscript. KG and HJS: contributed to the analysis and interpretation of the data. JLK: provided logistical support, critically revised the manuscript. All authors read and approved the final manuscript.

## Pre-publication history

The pre-publication history for this paper can be accessed here:

http://www.biomedcentral.com/1471-2482/12/24/prepub

## References

[B1] Magendie F Sur l’entree accidentelle de I’air dans les veines, sur la mort subite, qui en est l’effet; sur les moyens de prevenir cet accident et d'y remedier J Physiol Exp Pathol 1821 1 190

[B2] Albin MS Venous air embolism. A warning not to be complacent – we should listen to the drumbeat of history Anesthesiology 2011 115 626 629 10.1097/ALN.0b013e31822a6408 21799396

[B3] Mirski MA Lele AV Fitzsimmons L Toung TJ Diagnosis and treatment of vascular air embolism Anesthesiology 2007 106 164 177 10.1097/00000542-200701000-00026 17197859

[B4] Domaingue CM Anaesthesia for neurosurgery in the sitting position: a practical approach Anaesth Intensive Care 2005 33 323 331 15973914 10.1177/0310057X0503300307

[B5] Leonard IE Cunningham AJ The sitting position in neurosurgery – not yet obsolete! Br J Anaesth 2002 88 1 3 10.1093/bja/88.1.1 11881863

[B6] Michenfelder JD Martin JT Altenburg BM Rehder K Air embolism during neurosurgery: An evaluation of right atrial catheters for diagnosis and treatment JAMA 1969 208 1353 1358 10.1001/jama.1969.03160080017004 5818793

[B7] Feberowski LW Black S Mickle JP Incidence of venous air embolism during craniectomy for craniosynostosis repair Anesthesiology 2000 92 20 23 10.1097/00000542-200001000-00009 10638894

[B8] Souders JE Pulmonary air embolism J Clin Monit 2000 16 375 383 10.1023/A:1011455701892 12580220

[B9] Mason RJ Greene K Voelker DR Surfactant protein A and surfactant protein D in health and disease Am J Physiol Lung Cell Mol Physiol 1998 275 L1 L13 10.1152/ajplung.1998.275.1.L1 9688929

[B10] Hermans C Bernard A Lung epithelium-specific proteins. Characteristics and potential applications as markers Am J Respir Crit Care Med 1999 159 646 678 9927386 10.1164/ajrccm.159.2.9806064

[B11] Chida S Phelps DS Soll RF Taeusch HW Surfactant proteins and anti-surfactant antibodies in sera from infants with respiratory distress syndrome with and without surfactant treatment Pediatrics 1991 88 84 89 2057277

[B12] Bernard A Marchandise FX Depelchin S Lauwerys R Sibille Y Clara cell protein in serum and bronchoalveolar lavage Eur Respir J 1992 5 1231 1238 1486970

[B13] Hermans C Knoops B Wiedig M Salane K Toubeau G Falmange P Bernard A Clara cell protein as a marker of Clara cell damage and bronchoalveolar blood barrier permeability Eur Respir J 1999 13 1014 1021 10.1034/j.1399-3003.1999.13e14.x 10414398

[B14] Pittet JF Mackersie RC Martin TR Matthay MA Biological markers of acute lung injury: prognostic and pathogenetic significante Am J Respir Crit Care Med 1997 155 1187 1205 9105054 10.1164/ajrccm.155.4.9105054

[B15] Cheng IW Ware LB Greene KE Nuckon TJ Eisner MD Matthay MA Prognostic value of surfactant proteins A and D in patients with acute lung injury Crit Care Med 2003 31 20 27 10.1097/00003246-200301000-00003 12544988

[B16] Tzouvelekis A Koulitasis G Anevlavis S Bouros D Serum biomarkers in interstitial lung diseases Respir Res 2005 6 78 102 10.1186/1465-9921-6-78 16042760 PMC1215520

[B17] Papadopoulos G Kuhly P Brock M Rudolph JL Eyrich K Venous and paradoxical air embolism in the sitting position: a prospective study with transoesophageal echocardiography Acta Neurochir 1994 126 140 143 10.1007/BF01476424 8042546

[B18] Russell GB Graybeal JM Detection of venous air embolism: comparison of oxygenation and ventilation monitoring methods in dogs J Neurosurg Anesthesiol 1992 4 36 40 10.1097/00008506-199201000-00007 10147762

[B19] Nossum V Hjelde A Brubakk AO Small amounts of venous gas embolism cause delayed impairment of endothelial function and increase polymorphonuclear neutrophil infiltration Eur J Appl Physiol 2002 86 209 214 10.1007/s00421-001-0531-y 11990728

[B20] Barak M Katz Y Microbubbles: pathophysiology and clinical implications Chest 2005 128 2918 2932 10.1378/chest.128.4.2918 16236969

[B21] Determann RM Royakkers A Haitsma JJ Zhang H Slutsky AS Ranieri VM Schultz MJ Plasma levels of surfactant protein D and KL-6 for evaluation of lung injury in critically ill mechanically ventilated patients BMC Pulm Med 2010 10 6 10.1186/1471-2466-10-6 20158912 PMC2841652

[B22] Honda Y Kuroki Y Matsuura E Nagae H Takahashi H Akino T Abe S Pulmonary surfactant protein D in sera and bronchoalveolar lavage fluids Am J Respir Crit Care Med 1995 152 1860 1866 8520747 10.1164/ajrccm.152.6.8520747

[B23] Plotz FB Slutsky AS van Vucht AJ Heijnen CJ Ventilator-induced lung injury and multiple system organ failure: a critical review of facts and hypotheses Intensive Care Med 2004 30 1865 1872 10.1007/s00134-004-2363-9 15221129

[B24] Determann RM Wolthuis EK Choi G Bresser P Bernard A Lutter R Schultz MJ Lung epithelial injury markers are not influenced by use of lower tidal volumes during elective surgery in patients without preexisting lung injury Am J Physiol Lung Cell Mol Physiol 2008 294 L344 L350 18083770 10.1152/ajplung.00268.2007

[B25] Blair E Hickam JB The effect of change in body position on lung volume and intrapulmonary gas mixing in normal subjects J Clin Invest 1955 34 3 383 389 10.1172/JCI103086 14354008 PMC438639

[B26] Burns SM Egloff MB Ryan B Carpenter R Burns JE Effect of body position on spontaneous respiratory rate and tidal volume in patients with obesity, abdominal distension and ascites Am J Crit Care 1994 3 102 106 8167771

[B27] Kropski JA Fremont RD Calfee CS Ware LB Clara cell protein (CC16), a marker of lung epithelial injury, is decreased in plasma and pulmonary edema fluid from patients with acute lung injury Chest 2009 135 1440 1447 10.1378/chest.08-2465 19188556 PMC2716712

[B28] Bernard A Thielemans N Lauwerys R Vandeleene B Lambert AE The renal handling of protein 1 (Clara cell protein): effect of age, sex and renal dysfunction Contrib Nephrol 1993 101 66070 10.1159/000422110 8467690

[B29] Itoh Y Ishii S Okutani R Asano Y Kawai T Protein 1: its purification and application in clinical medicine J Clin Lab Anal 1993 7 394 400 10.1002/jcla.1860070614 8277362

[B30] Bernard A Dumont X Roels H Lauwerys R Dierynck I DeLey M Stroobant V de Hoffmann E The molecular mass and concentrations of protein 1 or Clara cell protein in biological fluids: a reappraisal Clin Chim Acta 1993 223 189 191 10.1016/0009-8981(93)90077-H 8143367

[B31] Gale T Leslie K Anaesthesia for neurosurgery in the sitting position J Clin Neurosci 2004 11 693 696 10.1016/j.jocn.2004.05.007 15337126

[B32] Hlastala MP Robertson HT Ross BK Gas exchange abnormalities produced by venous gas emboli Respirat Physiol 1979 36 1 17 10.1016/0034-5687(79)90011-2 217052

